# FDX1 can Impact the Prognosis and Mediate the Metabolism of Lung Adenocarcinoma

**DOI:** 10.3389/fphar.2021.749134

**Published:** 2021-10-08

**Authors:** Zeyu Zhang, Yarui Ma, Xiaolei Guo, Yingxi Du, Qing Zhu, Xiaobing Wang, Changzhu Duan

**Affiliations:** ^1^ Department of the First Clinical Medicine, Chongqing Medical University, Chongqing, China; ^2^ Department of Medical Oncology, Beijing Hospital, National Center of Gerontology, Institute of Geriatric Medicine, Chinese Academy of Medical Sciences, Beijing, China; ^3^ Binzhou Polytechnic, Binzhou, China; ^4^ State Key Lab of Molecular Oncology, National Cancer Center/National Clinical Research Center for Cancer/Cancer Hospital, Chinese Academy of Medical Sciences and Peking Union Medical College, Beijing, China; ^5^ Department of Clinical Laboratory, Beijing Friendship Hospital, Capital Medical University, Beijing, China; ^6^ Department of Cell Biology and Genetics, Medicine and Cancer Research Center, Chongqing Medical University, Chongqing, China

**Keywords:** lung cancer, inflammatory response, mitochondria electron transport chain, risk signature, metabolism

## Abstract

**Background:** Lung cancer has emerged as one of the most common cancers in recent years. The mitochondrial electron transport chain (ETC) is closely connected with metabolic pathways and inflammatory response. However, the influence of ETC-associated genes on the tumor immune response and the pathogenesis of lung cancer is not clear and needs further exploration.

**Methods:** The RNA-sequencing transcriptome and clinical characteristic data of LUAD were downloaded from the Cancer Genome Atlas (TCGA) database. The LASSO algorithm was used to build the risk signature, and the prediction model was evaluated by the survival analysis and receiver operating characteristic curve. We explored the function of FDX1 through flow cytometry, molecular biological methods, and liquid chromatography–tandem mass spectrometry/mass spectrometry (LC–MS/MS).

**Results:** 12 genes (*FDX1*, *FDX2*, *LOXL2*, *ASPH*, *GLRX2*, *ALDH2*, *CYCS*, *AKR1A1*, *MAOB*, *RDH16*, *CYBB*, and *CYB5A*) were selected to build the risk signature, and the risk score was calculated with the coefficients from the LASSO algorithm. The 1-year, 3-year, and 5-year area under the curve (AUC) of ROC curves of the dataset were 0.7, 0.674, and 0.692, respectively. Univariate Cox analysis and multivariate Cox regression analysis indicated that the risk signature is an independent risk factor for LUAD patients. Among these genes, we focused on the *FDX1* gene, and we found that knockdown of FDX1 neither inhibited tumor cell growth nor did it induce apoptosis or abnormal cell cycle distribution. But FDX1 could promote the ATP production. Furthermore, our study showed that FDX1 was closely related to the glucose metabolism, fatty acid oxidation, and amino acid metabolism.

**Conclusion:** Collectively, this study provides new clues about carcinogenesis induced by ETC-associated genes in LUAD and paves the way for finding potential targets of LUAD.

## Introduction

There have been an increasing number of new cancer cases every year. Lung cancer is one of the most morbidity diseases among all cancers, with a proportion as high as 11.6% (Bray et al., 2018; Barta et al., 2019). Lung cancer has a poor prognosis and is one of the deadly cancers in humans; over one-half of patients die within 1 year of diagnosis (Zappa and Mousa, 2016; Kimura et al., 2018; Duma et al., 2019). Non–small-cell lung carcinoma (NSCLC) accounts for 80–85% of all lung cancers (Sher et al., 2008). Lung adenocarcinoma (LUAD) is the most common histological type of NSCLC, accounting for 50% of NSCLCs (Bi et al., 2020; Zhang et al., 2020).

Metabolism plays an important role in carcinogenesis, and in recent years, it has been leveraged for tumor treatments (Biswas, 2015; Vander Heiden and DeBerardinis, 2017; Kumar and Misra, 2019). Multiple studies have shown that metabolism and cancer are closely related; currently, metabolites are important targets in the treatment of cancer (Chae and Kim, 2018; Dinges et al., 2019; Huang et al., 2021). The mitochondria electron transport chain locates in the inner membrane of the mitochondria and is composed of four complexes, including complexes I, II, III, and ATP synthase (Chen and Butow, 2005). Mutations in mitochondria DNA (mDNA) have been identified in several tumor types (Larman et al., 2012; Bonora et al., 2021; Kopinski et al., 2021). For example, mitochondrial fission regulator 2 (MTFR2) was a biomarker for diagnosis and poor prognosis in LUAD (Chen et al., 2021).

The mitochondria in tumor cells contain the rich protein network, and the interaction between these proteins is very important for initiating an antitumor immune response. Studies have reported that the enzyme RIPK3 can regulate the activity of mitochondrial enzyme PGAM5 and trigger the expression of inflammatory cytokines in NKT cells, thus playing a dual role in the development of autoimmune diseases and the destruction of tumor cells (Kang et al., 2015). The intensity of the inflammatory response and multiple metabolic processes in patients with lung cancer activate each other and jointly promote the progression of the disease. As the inflammatory response intensifies, the metabolic dysfunction becomes more obvious. Studies have found that the serum levels of PCT and IL-6 after lung cancer surgery are higher than those before surgery. Combined detection of PCT and IL-6 can be an effective means to identify complications after lung cancer surgery (Zhu et al., 2019). Therefore, exploring mitochondrial metabolism has important practical significance for tumor progression, tumor prognosis, tumor treatment, and many other aspects.

In this study, we investigated the role of the ETC genes in the overall survival of LUAD using LUAD transcriptome expression data from the TCGA database. Next, we constructed cells with FDX1 knocked down and explored the changes in cellular biological functions, including the proliferation and apoptosis rates, the cell cycle distribution, and cellular metabolism.

## Methods

### Dataset Analysis

The RNA-sequencing transcriptome and clinical characteristic data of the LUAD cohort which contained 535 LUAD patients and 59 normal samples were downloaded from the TCGA database (https://cancergenome.nih.gov/). Mitochondria ETC genes were reported (Supplementary Table S1).

### Prognostic Risk Signature Prediction and Calculation

Univariate Cox analysis was performed to evaluate the correlation between ETC genes and overall survival (OS). A risk score for each patient was calculated as the sum of each gene’s score, which was obtained by multiplying the expression of each gene and its coefficient. The cohort was divided into high- and low-risk groups based on the median value of the risk scores. The difference of OS between high- and low-risk groups was calculated by the Kaplan–Meier method with a two-sided log-rank test. The receiver operating characteristic (ROC) curve was constructed to evaluate the prediction accuracy of the prognostic model.

The chi-square test was performed to compare the distribution of clinicopathological parameters between high- and low-risk groups. Heatmaps were used to visualize the difference with heatmap R package. Univariate and multivariate Cox regression analyses were used to identify the independent prognostic factors for the cohort. The survival difference between high-risk and low-risk groups stratified by age, gender, stage, and TNM was further evaluated.

### Single Gene Bioinformatics Analysis

The survival curve was plotted through the online tool PrognoScan (http://dna00.bio.kyutech.ac.jp/PrognoScan/index.html). The Go enrichment analysis was performed using clusterProfiler package in R environment. GSEA was carried out using the curated gene sets “c2.cp.kegg.v7.4.symbols.gmt” to illustrate the different enriched terms between the high and low FDX1 expression groups.

### Cell Culture

A549 cell line was obtained from the National Infrastructure of Cell Line Resource (Shanghai, China). The A549 cell line was cultured in RPMI 1640 containing 10% fetal bovine serum and penicillin (100 U/mL)/streptomycin (0.1 mg/ml), and cultured at 37°C in a humidified atmosphere containing 5% CO_2_.

### RNA Interference

Small interfering RNA was purchased from GenePharma (Suzhou, China). A549 cells were transfected with 50 nm FDX1 siRNAs using Lipofectamine RNAiMAX (Invitrogen/Thermo Fisher Scientific, Cat# 13778030). The knockdown efficiency was evaluated by quantitative RT-PCR after 72 h of transfection. The following siRNA sequences were used: FDX1-1-F, CAU​CUU​UGA​AGA​UCA​CAU​Att; FDX1-1-R, UAU​GUG​AUC​UUC​AAA​GAU​Gag; FDX1-2-F, GCA​UAU​GGA​CUA​ACA​GAC​Att; and FDX1-2-R, UGU​CUG​UUA​GUC​CAU​AUG​Cca.

### RT-PCR

Total RNA was extracted from the cells using TRIzol reagent (Thermo Scientific, Cat# 15596018). Then, the RNA was quantified using Nanodrop. The RNA was reverse-transcribed into DNA using a PrimeScript RT Reagent Kit (Takara, Cat# RR036A) according to the manufacturer’s protocol. The primers were as follows: FDX1-F, TTC​AAC​CTG​TCA​CCT​CAT​CTT​TG; FDX1-R, TGC​CAG​ATC​GAG​CAT​GTC​ATT; GAPDH-F, GGA​GCG​AGA​TCC​CTC​CAA​AAT; and GAPDH-R, GGC​TGT​TGT​CAT​ACT​TCT​CAT​GG.

### Cell Growth Assays

The CCK-8 agent (Dojindo, Cat# CK04) was used to detect cell viability. A549 cells were plated in 96-well plates at 2000 cells per well. Then, CCK-8 was added to each well every 24 h for 5 days according to the manufacturer’s protocol. The absorbance value was detected using a microplate reader (Bio-Rad Laboratories; Hercules, CA, USA) at 450 nm.

### Flow Cytometry

Both apoptosis and cell cycle analysis were analyzed on a flow cytometer (Beckman Coulter) according to the manufacturer’s instructions. Apoptosis was determined using the Apoptosis Detection Kit (Dojindo, Kumamoto, Japan) and analyzed with FlowJo v10 (FlowJo, LLC). Cell cycle analysis was performed using the Cell Cycle and Apoptosis Analysis Kit (Beyotime; Jiangsu, China) and analyzed by Modfit LT 3.2 software (Verity Software House; www.vsh.com; Topsham, ME).

### ATP Measurement

ATP levels were measured with an ATP assay kit (Beyotime Biotechnology, Cat# S0027) according to the manufacturer’s instructions. In brief, 20 μl cell sample was mixed with 100 µl ATP detection working fluid; the luminescence was measured using a multi-scan spectrum (BioTEK H1MFD). The concentration of ATP in the sample was calculated according to the standard curve. In order to eliminate the error caused by the difference of the protein amount in sample preparation, the concentration of ATP is converted to nmol/mg.

### Metabolic Profiling

SiFDX1 cells and control cells were cultured in 10-cm plates. The metabolites were extracted using prechilled 80% (v/v) HPLC-grade methanol after culturing for 72 h and then incubated overnight at −80°C. The tubes were centrifuged for 20 min at 14,000 g, and the supernatant was transferred to new tubes. Then, the protein precipitation was used to be quantified by the BCA method. The supernatant was concentrated by a vacuum centrifugal concentrator for 1 h. Furthermore, the metabolites were redissolved according to the protein concentrations. The polar metabolites were detected by liquid chromatography–tandem mass spectrometry/mass spectrometry (LC–MS/MS). Data were acquired in both positive (ESI+) and negative (ESI-) modes. All the data were used for the statistical analysis. T-test results were evaluated with Excel. MetaboAnalyst (www.metaboanalyst.ca) was used to determine the heatmap and differential metabolic pathways.

### Statistical Analysis

All statistical analyses were performed using R software (version 3.5.1). Wilcoxon’s test was used to compare the expression of ETC genes between LUAD and adjacent normal tissues. The relationship between ETC genes and the clinicopathological characteristics of LUAD patients was analyzed using the Kolmogorov–Smirnov test. Using the “km” method in the R package “ConsensusClusterPlus,” we classified 535 LUAD patients into different subtypes. The median risk score was used as a cutoff value to classify patients into a high-risk group and a low-risk group, and the Kaplan–Meier method was used to analyze the difference in overall survival (OS) between the high- and low-risk groups. The chi-square test was used to compare the relationship between the risk score and clinicopathological variables. Cox univariate and multivariate analyses of the relationship between clinicopathological variables and risk scores were performed. *p* < 0.05 was considered to indicate a statistically significant difference.

## Results

### Expression Pattern and Risk Signature of ETC Genes in LUAD.

Mitochondrial ETC plays a pivotal role in cell energy metabolism. The occurrence and development of lung adenocarcinoma are closely linked to cell metabolism, and we hope to explore the impact of mitochondrial ETC gene expression in LUAD. We downloaded the ETC genes from online database AmiGO2 (http://amigo.geneontology.org/amigo). LUAD cancer tissues and normal lung tissues were compared to explore the expression level of mitochondria ETC genes (Supplementary Table S2). A total of 47 differential expression genes (DEGs) were identified with high expression of 34 genes and low expression of 13 genes in LUAD tissues (Supplementary Figure S1, Supplementary Table S2).

**FIGURE 1:**
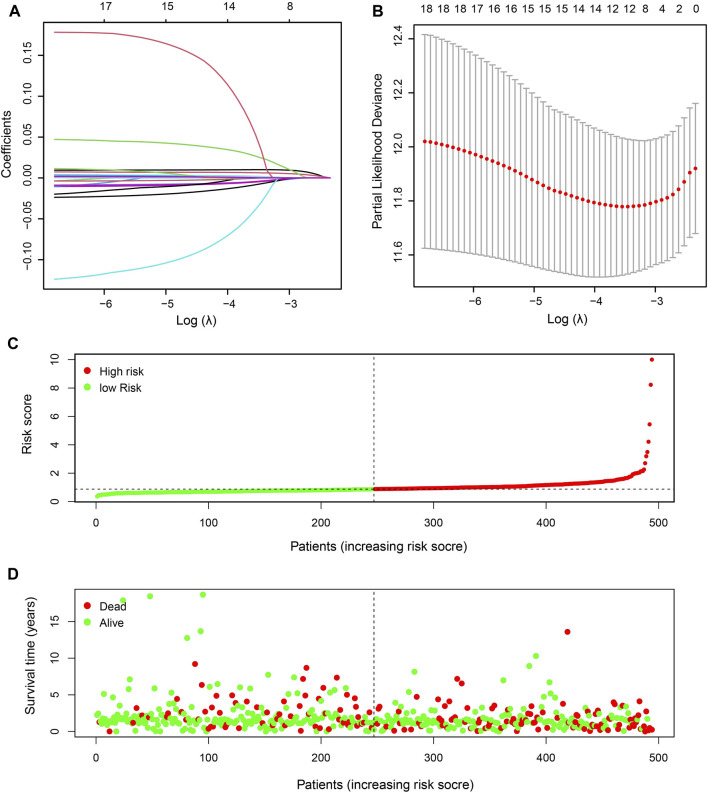
Identification of prognostic ETC-associated genes. **(A)**. The LASSO coefficient distribution map of the 12 ETC genes in LUAD patients. **(B)**. The partial likelihood deviance plot based on the LASSO model. **(C)**. The risk score distribution of the 12-gene risk signature model in LUAD patients. **(D)**. The survival status of patients in high-risk and low-risk groups of LUAD patients.

**FIGURE 2:**
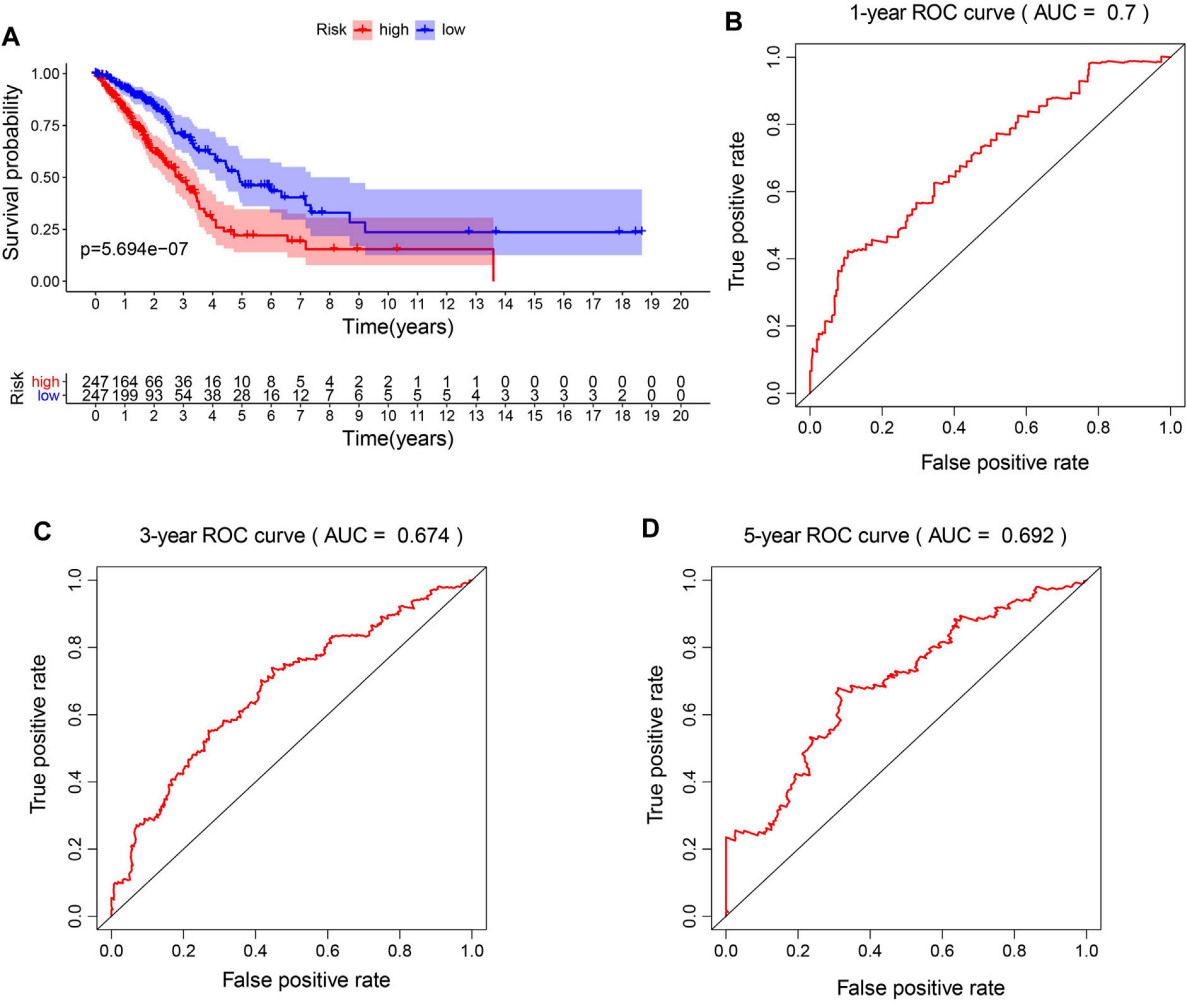
Prognostic accuracy of the risk model. **(A)**. The survival analysis of high-risk signature and low-risk signature in LUAD patients. **(B–D)**. The 1-year, 3-year, and 5-year area under the curve (AUC) of ROC curves.

To better explore the relationship between mitochondria ETC genes and clinical outcomes in LUAD patients, univariate Cox regression analysis was performed to evaluate the prognostic role of ETC genes in LUAD (Supplementary Table S3). In order to predict the clinical outcomes of LUAD with ETC genes, we applied the least absolute shrinkage and selection operator (LASSO) Cox regression algorithm to the differential expression ETC genes to construct the prognostic signature. According to the partial likelihood deviance and lambda values, 12 genes (*FDX1*, *FDX2*, *LOXL2*, *ASPH*, *GLRX2*, *ALDH2*, *CYCS*, *AKR1A1*, *MAOB*, *RDH16*, *CYBB*, and *CYB5A*) were selected to build the risk signature, and the risk score was calculated with the coefficients from the LASSO algorithm (Figures 1A,B) (Supplementary Table S4). According to the median risk score, LUAD patients were divided into low- and high-risk groups to investigate the prognostic role of the risk signature (Figures 1C,D). The results indicated that the high-risk group had a worse survival (Figure 2A). Moreover, a receiver operating characteristic (ROC) curve was generated to assess the predictive value of the constructed model. The 1-year, 3-year, and 5-year area under the curve (AUC) of ROC curves of the TCGA-LUAD dataset were 0.7, 0.674, and 0.692, respectively (Figures 2B–D).

### The Risk Score was an Independent Prognostic Factor in LUAD.

**FIGURE 3:**
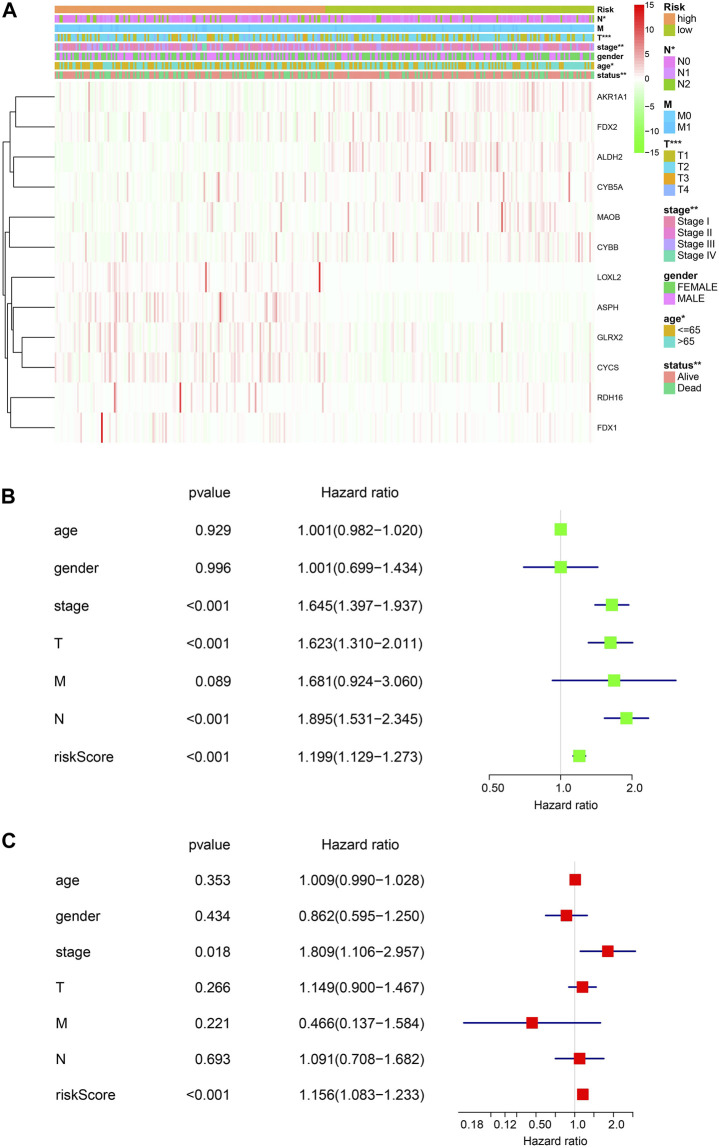
Risk signature of ETC genes in LUAD. **(A)**. The associations of risk signature and clinicopathological features (T, N, M, tumor stage, gender, age, and the survival status). **(B, C)**. Univariate and multivariate Cox analyses of the clinicopathological features (age, gender, stage, T, N, and M) and risk signature of overall survival in LUAD patients.

Next, the associations of the risk signature and clinicopathological features were evaluated, and we discovered that significant difference was observed of N stage, T stage, stage, age, and survival status between the two risk groups, while no significant difference was found of M and gender between the two risk groups (Figure 3A). Furthermore, we performed univariate and multivariate Cox regression analyses to determine whether the risk signature was an independent prognostic indicator. Univariate Cox analysis showed that the risk score, stage, and T and N status were correlated with the OS (Figure 3B). Multivariate Cox regression analysis indicated that the risk score was correlated with the OS (Figure 3C). These results suggested that the risk signature is an independent risk factor for LUAD patients.

### *FDX1* Plays a Pivotal Role in LUAD.

**FIGURE 4:**
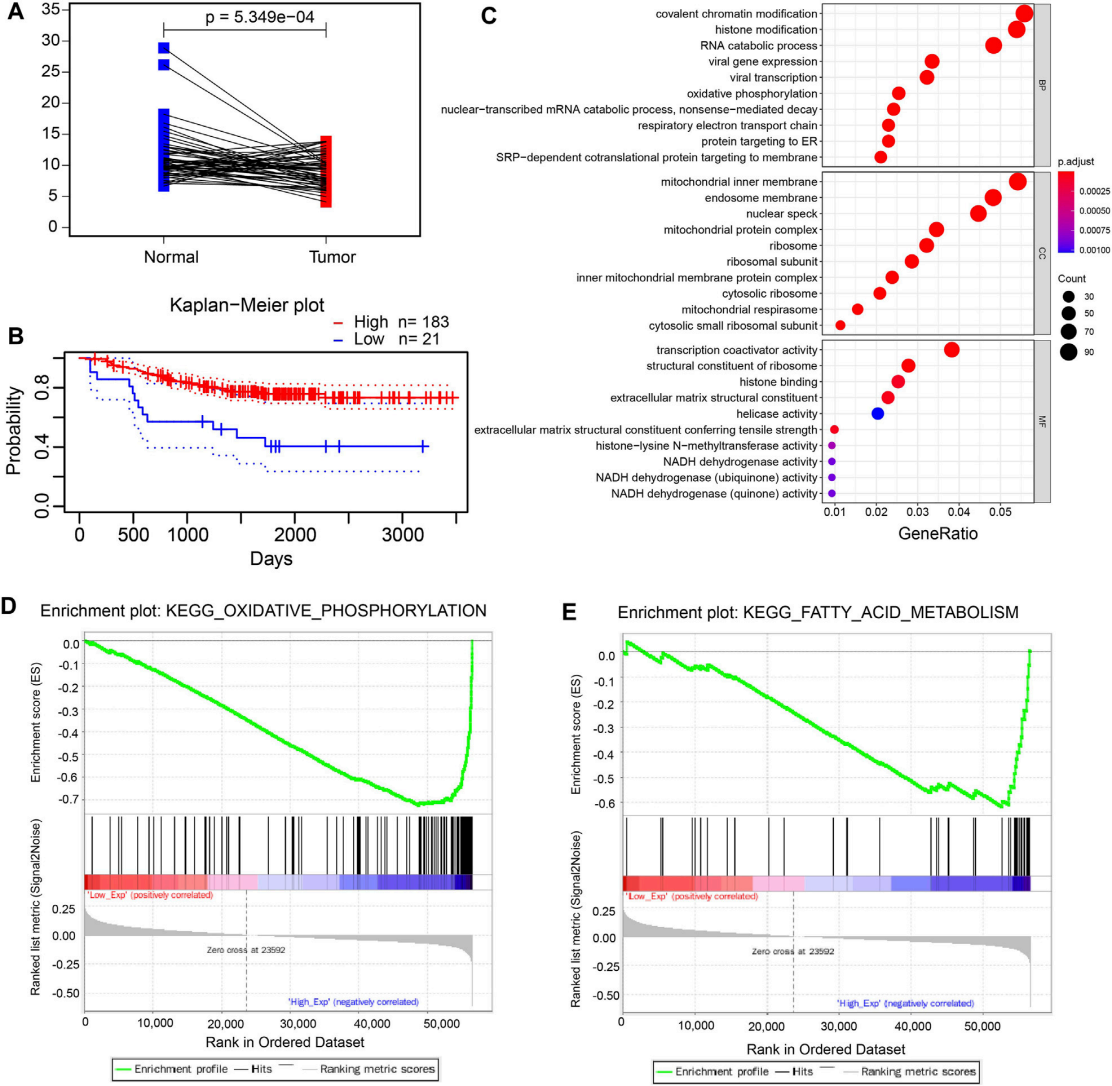
FDX1 plays a pivotal role in LUAD. **(A)**. The mRNA level of paired LUAD tumor tissues and adjacent normal tissues in the TCGA-LUAD dataset. **(B)**. The Kaplan–Meier plot of low expression and high expression of FDX1 in LUAD patients using the ProgScan online tool. c. The GO enrichment analysis of FDX1. **(D, E)**. The GSEA analysis of low expression of FDX1.

*FDX1* encodes a small iron–sulfur protein that participates in the reduction in mitochondrial cytochrome and the synthesis of various steroid hormones (Sheftel et al., 2010; Strushkevich et al., 2011). Also, FDX1 can augment the copper-dependent cell death induced by elesclomol and may provide a new idea for increasing the efficacy of several cancer-targeting agents (Tsvetkov et al., 2019). Next, we focused on *FDX1* and explored its function in LUAD. The mRNA expression level was significantly decreased in the tumor tissues compared to adjacent normal tissues in the TCGA-LUAD dataset (Figure 4A). Also, we discovered that LUAD patients with the lower expression of FDX1 had a worse prognosis using the online analysis tool PrognoScan (http://dna00.bio.kyutech.ac.jp/PrognoScan/index.html) (Figure 4B), which suggested that FDX1 played a pivotal role in LUAD patients. Moreover, the GO enrichment analyses revealed that FDX1 was mainly associated with oxidative phosphorylation, respiratory electron transport chain, and mitochondrial protein complex (Figure 4C). In addition, GSEA enrichment analysis was performed to explore the underlying mechanism of FDX1, and the analysis suggested that low expressed FDX1 was correlated with fatty acid metabolism and oxidative phosphorylation metabolism (Figures 4D,E). Furthermore, we investigated the function of FDX1 in the online database (http://bioinfo.life.hust.edu.cn/GSCA/#/) and found that FDX1 had significant effect on immune-associated cells, such as dendritic cells, macrophage, and iTreg cells (Supplementary Figure S3).

### Knockdown of FDX1 Neither Inhibited Tumor Cell Growth Nor Did It Induce Apoptosis or Cell Cycle Arrest.

**FIGURE 5:**
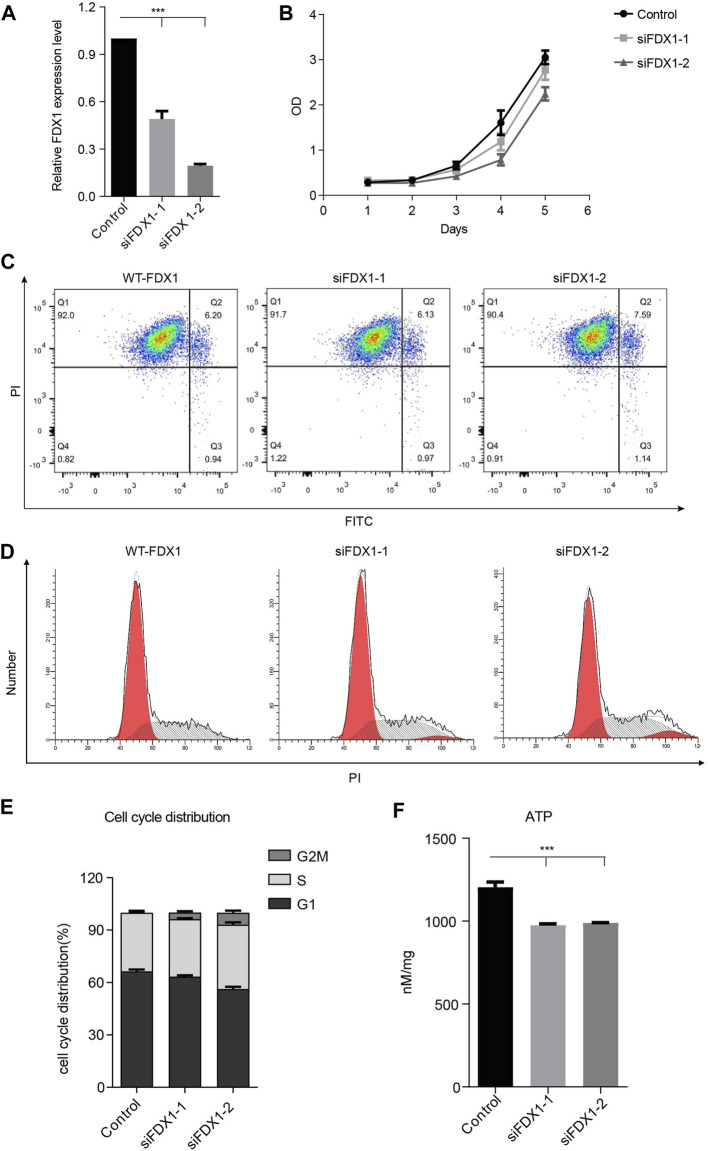
Biological function of FDX1 in A549 cells. **(A)**. Expression of FDX1 in A549 cells transfected with si-FDX1. **(B)**. The cell growth of the FDX1 deficiency group and control group was assessed by the CCK-8 assay. **(C)**. The apoptotic rate of the control group and FDX1 deficient group. **(D)**. PI staining was used to detect the cell cycle distribution of the control group and FDX1-deficient group. **(E)**. The cell cycle distribution of FDX1-knockdown cells and FDX1-wild-type cells. **(F)**. ATP levels of FDX1-deficient cells and wild-type A549 cells.

To investigate whether FDX1 deficiency affects the growth of tumor cells, we constructed the FDX1-deficient cells using small interference RNA and determined the knockdown efficiency through qPCR. We found that the siRNAs significantly inhibited the expression of FDX1 (Figure 5A). However, knockdown of FDX1 did not change the cell viability through assay (Figure 5B). We further investigated the influence of FDX1 on apoptosis, and the effects of two siRNA groups were not significantly different (Figure 5C). Next, we determined the cell cycle changes in FDX1-deficient cells (KD-FDX1) and FDX1 wild-type cells (WT-FDX1). Knocking down of FDX1 did not induce G2/M cell cycle arrest significantly (Figures 5D,E). These data confirmed that knockdown of FDX1 neither affected cell growth nor did it induce apoptosis or cell cycle arrest. To confirm the effect of FDX1 deficiency on ATP levels, we measured the production of ATP in FDX1-wild-type cells and FDX1-knockdown cells, and the results showed that the ATP concentration of the FDX1 deficiency group was lower than that of the control group (Figure 5F). These data inspired us to explore more about the aberrant metabolism caused by FDX1 deficiency.

### Metabolic Profiling of Tumor Cells With FDX1 Deficiency.

**FIGURE 6:**
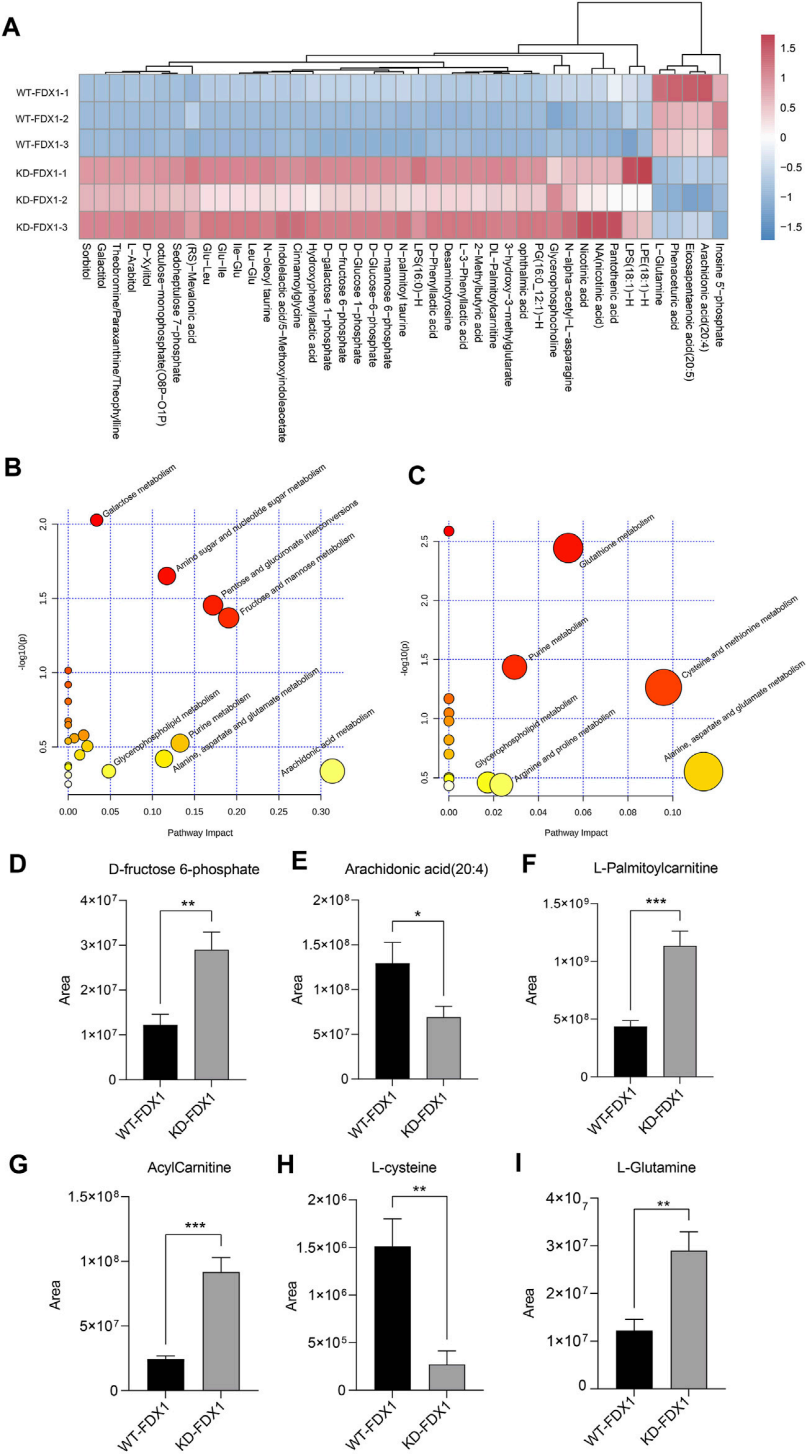
Metabolic profiling of tumor cells with FDX1 deficiency. **(A)**. The heatmap of differential metabolites in WT-FDX1 and KD-FDX1 cells in the negative ion mode. **(B, C)**. Pathway enrichment analysis of the differential metabolites in both the negative and positive ion modes. **(D–I)**. The levels of D-fructose 6-phosphate, arachidonic acid (20:4), L-palmitoylcarnitine, acylcarnitine, L-cysteine, and L-glutamine in WT-FDX1 and KO-FDX1 cells. The data are presented as the mean ± SD. *p < 0.01, **p < 0.001 and ***p < 0.0001 (*t*-test).

To further explore the metabolic changes caused by FDX1 deficiency, we performed nontargeted metabolomic analysis on WT-FDX1 cells and KD-FDX1 cells. The data showed that 10 metabolites and 5 metabolites were downregulated in positive and negative modes, respectively, with a threshold value of *p* < 0.05 and a ratio of WT-FDX1 cells to KD-FDX1 cells <0.6 (Supplementary Figure S2, Figure 6A). In addition, 79 metabolites and 38 metabolites were upregulated in positive and negative modes, respectively, with a threshold value of *p* < 0.05 and a ratio of WT-FDX1 cells to KD-FDX1 cells >2 (Supplementary Figure S2, Figure 6A). Differential metabolite levels were further analyzed using MetaboAnalyst (www.metaboanalyst.ca). Several pathways were identified in the negative mode, including amino sugar and nucleotide sugar metabolism, fructose and mannose metabolism, fatty acid degradation, and alanine, aspartate, and glutamate metabolism (Figure 6B). Thiamine metabolism, glutathione metabolism, and fatty acid degradation pathways were changed dramatically in the positive mode (Figure 6C).

Among these metabolites, fructose 6-phosphate was increased dramatically after FDX1 knockdown, one of the intermediate metabolites of glucose that affect downstream factors in the glycolysis and the pentose phosphate pathways (Figures 6D,E). In addition, long-chain fatty acids, including arachidonic acid (20:4), were decreased in FDX1-knockdown cells. In addition, acylcarnitine and L-palmitoylcarnitine were increased significantly in cells with FDX1 deficiency (Figures 6F,G). These data suggested that knockdown of FDX1 promoted fatty acid oxidation. Moreover, L-cysteine and L-glutamine metabolites involved in amino acid metabolism were affected by FDX1 deficiency (Figures 6H,I). Taken together, these data suggested that FDX1 deficiency led to metabolic changes, especially in glucose metabolism, fatty acid oxidation, and amino acid metabolism.

## Discussion

Over the years, the most prevalent causes of death have changed. Several studies have reported that cancer is the leading cause of death (Heron and Anderson, 2016), and lung cancer is one of the most common causes of death by cancer (Bray et al., 2018). For this reason, researchers have carried out a variety of studies on LUAD to obtain a better understanding of it. In this study, we identified the ETC genes that correlated closely to the overall survival of LUAD patients. The prognostic model was constructed by the Lasso Cox regression model. The LUAD patients were classified into two groups: low-risk group and high-risk group, and the high-risk group was associated with poor prognosis. Moreover, the univariate and multivariate Cox analyses suggested that the ETC-associated gene signature was an independent prognostic factor for predicting the outcome of LUAD patients.

We constructed the risk signature using the 12 genes. Among them, aspartate β-hydroxylase (*ASPH*) plays a pivotal role in the malignant transformation of solid tumors by enhancing cell proliferation, migration, invasion and stimulation of angiogenesis, and immunosuppression (Ince et al., 2000; Huyan et al., 2014; Kanwal et al., 2020). A previous study showed that repression of aldehyde dehydrogenase 2 family (*ALDH2*) promotes lung tumor progression through accumulated acetaldehyde and DNA damage (Li et al., 2019). *CYBB*, a gene related to ETC and ferroptosis, associated signature could predict the 1-, 3-, and 5-year survival rates of LUAD patients (Wang et al., 2021). Also, mutation of the *CYBB* gene selectively affects macrophages and is associated with immune changes (Bustamante et al., 2011). *FDX1* and *FDX2*, the important metabolism-related genes, are closely related to mitochondrial cytochrome (Sheftel et al., 2010; Strushkevich et al., 2011; Shi et al., 2012). These data suggested those genes were associated with the changes of the inflammatory response/immune microenvironment and the prognosis of LUAD patients.

Furthermore, we explored the function of FDX1 in LUAD cell lines. To our knowledge, the detailed function of FDX1 has not yet been elucidated in LUAD. In this study, our data showed that KD-FDX1 cells and WT-FDX1 cells had no difference in terms of growth, apoptosis rate, and cell cycle distribution, while it was involved in other cellular aspects, including aberrant energetics, tumor-associated inflammation, and changes in the immune microenvironment (Hanahan and Weinberg, 2011; Boroughs and DeBerardinis, 2015).

Aberrant metabolism links closely to the immune microenvironment in tumor development. Also, an increasing amount of evidence suggests that the metabolism plays an important role in the development and progression of cancer (Garon et al., 2014; La Vecchia and Sebastián, 2020). We have identified that KD-FDX1 cells have a significant influence on glucose metabolism, fatty acid oxidation (FAO), and amino acid metabolism. Glucose and lactic acid metabolism were reported to be common abnormalities in lung cancer (Hensley et al., 2016; Chen et al., 2019). Our data revealed that KD-FDX1 cells had higher D-fructose 6-phosphate levels. A recent research work has shown that D-fructose 6-phosphate associated with PFKP may be a prognostic factor for lung cancer (Ganapathy-Kanniappan, 2020). In addition, several studies have suggested that fatty acid oxidation is abnormal in lung cancer cells, and FAO may regulate immune suppression through enabling lymph node metastasis formation (Liu et al., 2020; Li et al., 2021). In our study, we found that KD-FDX1 cells have higher L-palmitoylcarnitine and acylcarnitine levels at the same arachidonic acid (20:4) concentration. In other words, this finding suggested that FDX1 knockdown promotes FAO. Increasing evidence has shown that glutamine can fulfill the metabolic needs of lung cancer cells. This is the first time to reveal the association of FDX1 with fatty acid oxidation (FAO) and amino acid metabolism in LUAD using nontargeted metabolomics. There are still some limitations in our article. For example, the number of cell lines we have verified is limited, and further expansion of cell lines can increase the credibility of the article. In addition, the molecular mechanism of FDX1’s regulation of metabolism still needs to be further explored. Overall, in our results, we found that FDX1 knockdown may decrease glutamine, and we speculated that FDX1 was likely to be a potential target in lung cancer treatment (Vanhove et al., 2019).

## Conclusion

Taken together, we constructed an accurate prognosis model of LUAD patients. Moreover, we explored the effect of FDX1 knockdown in LUAD cells. Although there was no obvious effect on cell growth, apoptosis, or cell cycle distribution in the KD-FDX1 and WT-FDX1 cells, knockdown of FDX1 may significantly affect the metabolism. Our results indicated that knockdown of FDX1 mainly promoted glycolysis and fatty acid oxidation, and changed amino acid metabolism. This study provides new clues about the carcinogenesis induced by FDX1 in LUAD and paves the way for finding potential targets of LUAD.

## Data Availability

The original contributions presented in the study are included in the article/Supplementary Material; further inquiries can be directed to the corresponding authors.

## Supplementary Material

Supplementary Figure S1 Volcano plot of the mRNA expression of ETC-associated genes in the TCGA-LUAD dataset.

Supplementary Figure S2 Heatmap of differential metabolites in WT-FDX1 and KD-FDX1 cells in the positive ion mode.

Supplementary Figure S3 Correlation between FDX1 expression and immune infiltrates in LUAD.

## Abbreviations

AUC, area under curve; ETC, electron transport chain; FAO, fatty acid oxidation; FDX1, ferredoxin 1; LC–MS/MS, liquid chromatography–tandem mass spectrometry/mass spectrometry; LUAD, lung adenocarcinoma; NSCLC, non–small-cell lung carcinoma; ROC, receiver operating characteristic; SD, standard deviation.
