# Supplementary figures and images for: FDX1 can Impact the Prognosis and Mediate the Metabolism of Lung Adenocarcinoma
Source: Front Pharmacol. 2021 Oct 8;12:749134. doi: 10.3389/fphar.2021.749134 (PMC8531531; doi:10.3389/fphar.2021.749134)

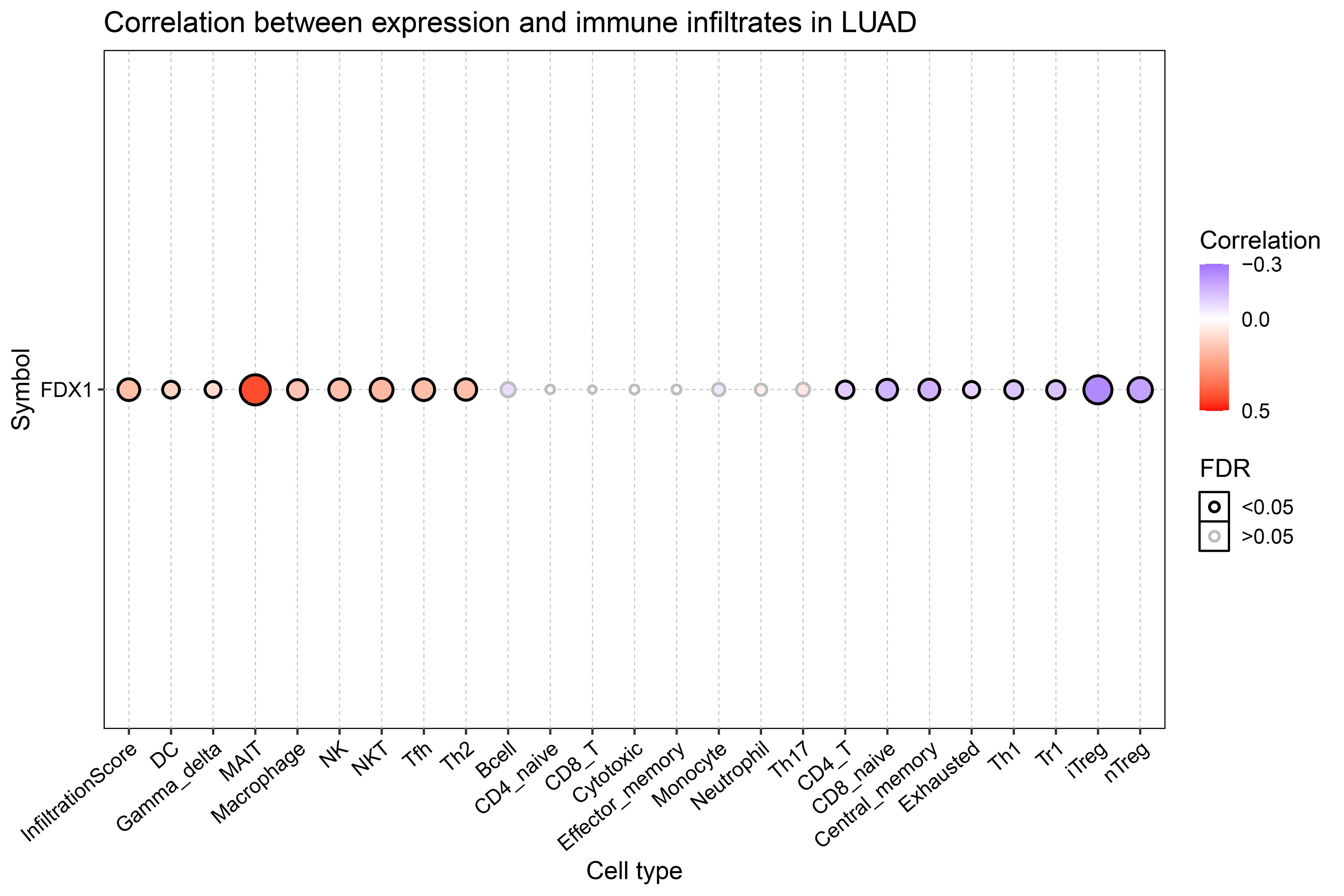

Supplement: Supplementary file 1 [file Image3.JPEG]

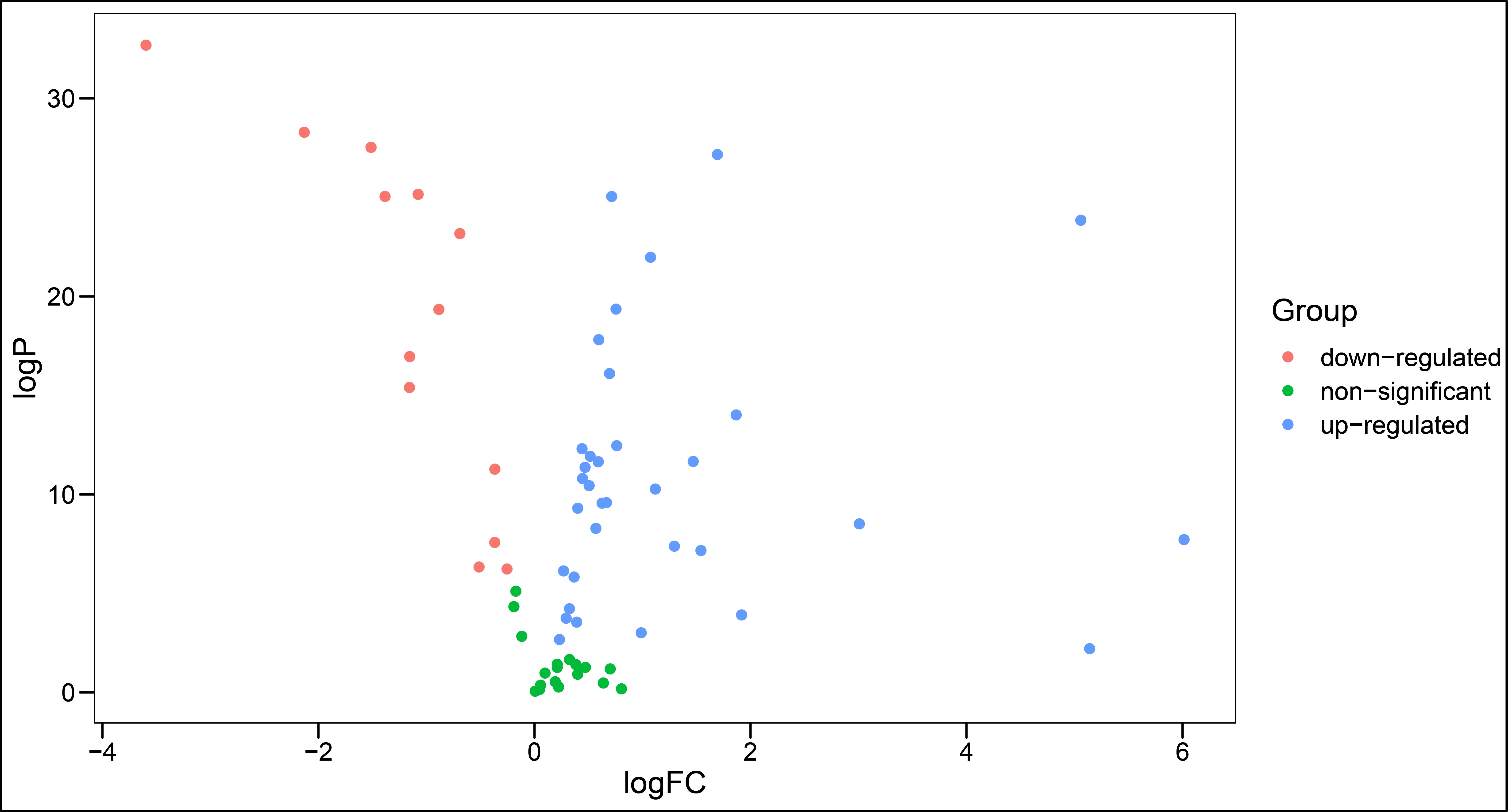

Supplement: Supplementary file 2 [file Image1.JPEG]

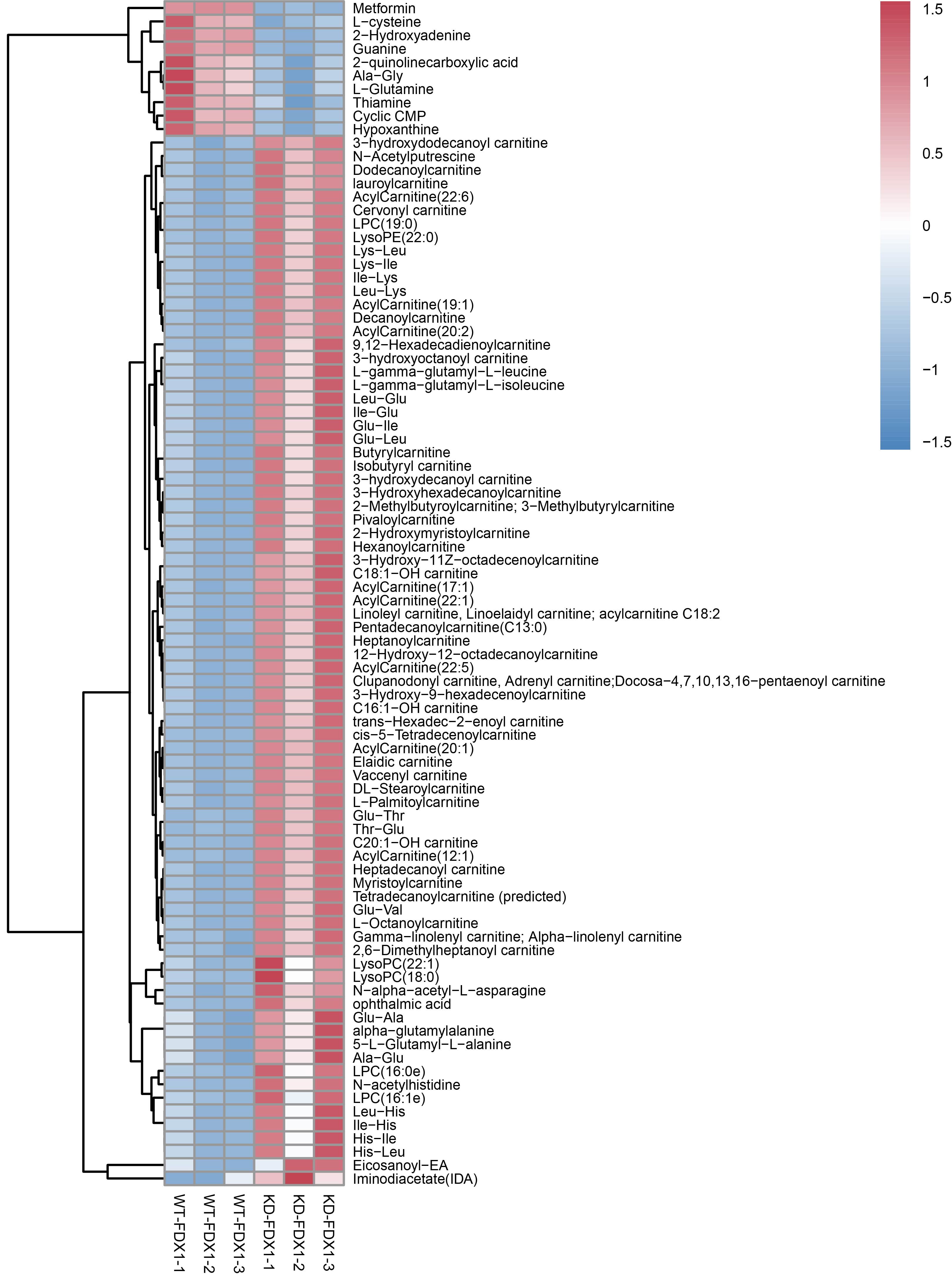

Supplement: Supplementary file 3 [file Image2.JPEG]
